# How Artificial Intelligence Can Enhance the Diagnosis of Cardiac Amyloidosis: A Review of Recent Advances and Challenges

**DOI:** 10.3390/jcdd11040118

**Published:** 2024-04-13

**Authors:** Moaz A. Kamel, Mohammed Tiseer Abbas, Christopher N. Kanaan, Kamal A. Awad, Nima Baba Ali, Isabel G. Scalia, Juan M. Farina, Milagros Pereyra, Ahmed K. Mahmoud, D. Eric Steidley, Julie L. Rosenthal, Chadi Ayoub, Reza Arsanjani

**Affiliations:** 1 Department of Cardiovascular Medicine, Mayo Clinic, Phoenix, AZ 85054, USA; 2 Division of Cardiovascular Imaging, Mayo Clinic, 5777 East Mayo Boulevard, Phoenix, AZ 85054, USA

**Keywords:** cardiac amyloidosis, artificial intelligence, deep-learning, convolutional neural networks

## Abstract

Cardiac amyloidosis (CA) is an underdiagnosed form of infiltrative cardiomyopathy caused by abnormal amyloid fibrils deposited extracellularly in the myocardium and cardiac structures. There can be high variability in its clinical manifestations, and diagnosing CA requires expertise and often thorough evaluation; as such, the diagnosis of CA can be challenging and is often delayed. The application of artificial intelligence (AI) to different diagnostic modalities is rapidly expanding and transforming cardiovascular medicine. Advanced AI methods such as deep-learning convolutional neural networks (CNNs) may enhance the diagnostic process for CA by identifying patients at higher risk and potentially expediting the diagnosis of CA. In this review, we summarize the current state of AI applications to different diagnostic modalities used for the evaluation of CA, including their diagnostic and prognostic potential, and current challenges and limitations.

## 1. Introduction

**Figure 1:**
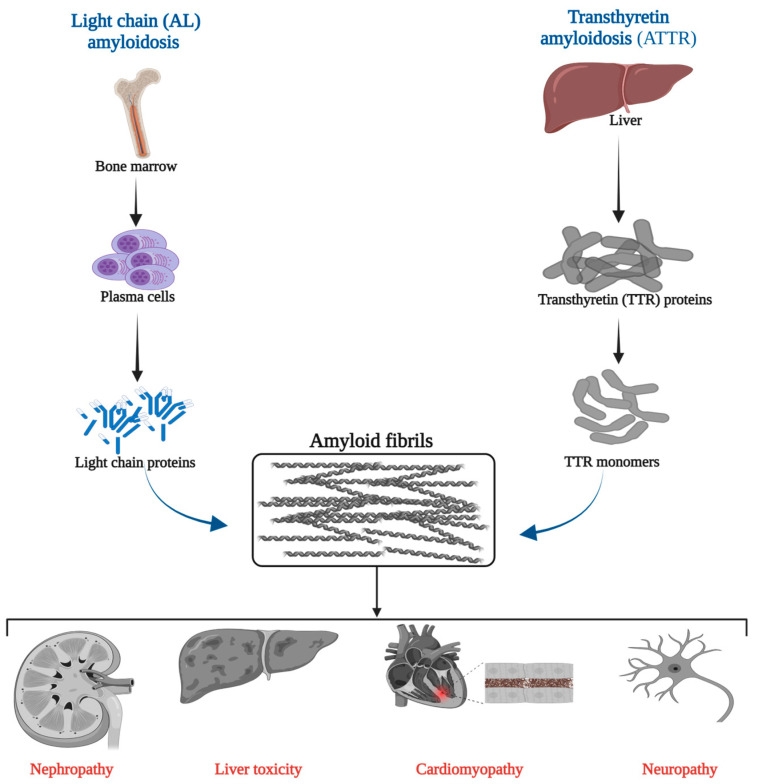
Pathophysiology of cardiac amyloidosis.

Cardiac amyloidosis (CA) is an underdiagnosed disease that carries a poor prognosis if not identified early [1]. The condition results from the misfolding and aggregation of insoluble proteins which then deposit extracellularly in myocardial tissue [2]. Two specific types affect the heart: immunoglobulin light chains (AL) and transthyretin amyloid protein (ATTR). While not all types of AL amyloidosis involve the heart, the occurrence of cardiac involvement significantly deteriorates the prognosis. For ATTR, the protein can be specified further, e.g., ATTRv for a variant, ATTRV30M for a specific mutation, or ATTRwt for the wild-type form [3,4]. The accumulation of these proteins in the myocardium disrupts the cardiac architecture and function, manifesting as a restrictive cardiomyopathy and progressive heart failure (Figure 1) [1,2,3,5].

Classification of CA subtypes is paramount in determining appropriate therapeutic strategies. The last decade has witnessed many advancements in disease-modifying therapies for each subtype. The pillar of managing AL-CA by hematologists is aimed to eliminate CD38+ plasma cells and to reduce amyloidogenic light chains, and in certain cases, autologous stem cell transplantation (ASCT) [6,7]. Until recently, ATTR-CA treatments had been targeted toward solely symptom management and disease-related complications. Currently, targeted options, namely tafamidis, stabilize the transthyretin tetramer to prevent its misfolding and the formation of amyloid fibrils [8,9]. There are numerous emerging therapies for ATTR-CA such as Patisiran, an RNA interference agent that inhibits the production of hepatic transthyretin which has exhibited convincing phase 3 clinical trial results, as well as additional ATTR silencers. CA exhibits considerable heterogeneity consisting of diverse clinical presentations; early in the disease process, manifestations may be very subtle. Clinical symptoms can be non-specific, including dyspnea, fatigue, and edema [10]. It is also an uncommon entity such that many cardiologists outside of tertiary institutions may not have extensive experience with its presentation. As a result, there is a propensity for late-stage diagnosis of CA [1,6]. Patients with CA are often evaluated by multiple providers before the establishment of the diagnosis, with one study quoting a correct diagnosis made in around 19% of cases after evaluation by cardiologists, a majority of diagnoses made one year after the presence of initial symptoms, and some with no unifying diagnosis for three years [6,11]. Overcoming these diagnostic challenges is critical and requires clinical expertise, the application of multiple diagnostic tools, and a multidisciplinary approach to care.

**Figure 2:**
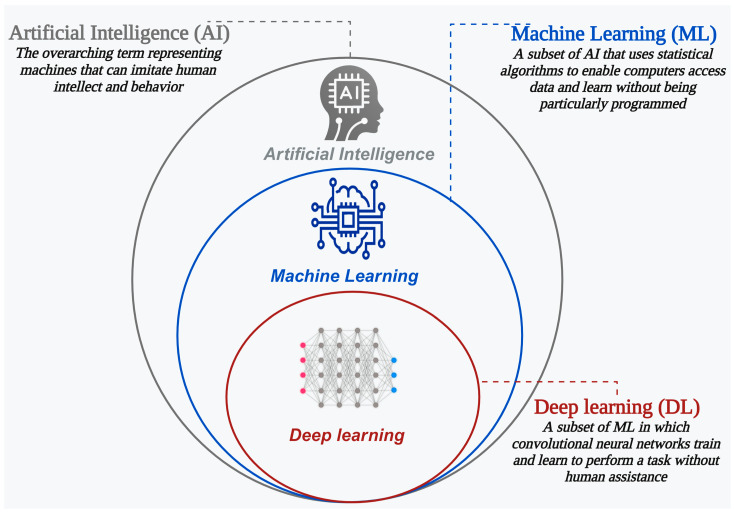
The hierarchical components of AI.

Artificial intelligence (AI) is the ability of machines to perform tasks that typically require human intelligence, such as learning, reasoning, and decision-making. It is an interdisciplinary field encompassing many applications and is increasingly being applied in medicine for both diagnosis and prognostication. Machine learning (ML) is a subset of AI that uses statistical algorithms to train models, allowing computers to learn from data without being explicitly programmed [12]. ML can be supervised, unsupervised, or reinforcement learning depending on the type and availability of data and feedback. Deep learning (DL) is a subset of machine learning that uses artificial neural networks to automatically identify and extract features from raw data such as images and text to make predictions or decisions [12,13]. Therefore, although the terms AI, ML, and DL may be used interchangeably, they are in fact hierarchical (Figure 2). Application of these AI tools is anticipated to revolutionize the practice of cardiovascular medicine and enhance patient care [14].

In the setting of the significant morbidity and mortality associated with CA, and the challenges and delays often associated with its diagnosis, applications of AI to modalities used in the evaluation for CA would carry significant clinical benefit. In this article, we summarize the current state of predictive AI analytic tools and their application to diagnostic modalities of CA. Their diagnostic and prognostic value will be reviewed, as well as potential associated challenges and limitations.

## 2. AI Applications to Electrocardiogram (ECG) in Cardiac Amyloidosis

**Figure 3:**
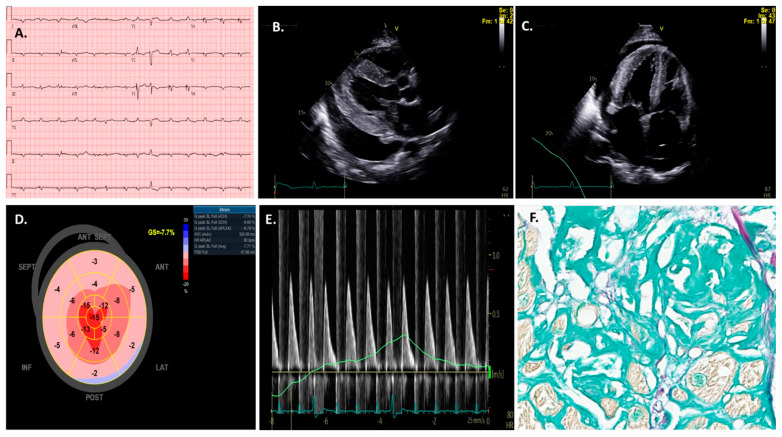
Traditional electrocardiogram (ECG) findings (**A**) in a patient with cardiac amyloidosis; note the low-voltage complexes with increased left-ventricular wall thickness on echocardiography (**B**,**C**). (**D**) depicts the traditional Bull’s-Eye graph of this disease; the apical longitudinal strain is preserved, but there is a substantial decrease in basal and medial segments. (**E**) shows another typical echocardiography feature of cardiac amyloidosis patients such as diastolic dysfunction. All the prior findings are critical for the diagnosis of this disease; however, confirmatory tests are needed following clinical suspicion. In this specific case, an endomyocardial biopsy (**F**) confirmed the diagnosis.

The electrocardiogram (ECG) remains the first-line and most widely utilized test for cardiac assessment (Figure 3A) [15,16]. ECG changes in CA reflect the multifaceted nature of this disease process [17], including myocardial infiltration, fibrosis, and conduction system dysfunction [18]. Arrhythmias and severe conduction abnormalities are prevalent in patients with CA, present in up to 40% of cases [19], with atrial fibrillation (AF) being the most frequent arrhythmia [20].

The most typical finding on ECG in CA is low voltage; however, it is present in 20–74% of cases, and its absence does not exclude the presence of CA [21,22]. While left-ventricular hypertrophy (LVH) is common in CA, some patients with CA may less commonly exhibit it on ECG [23,24]. CA is also commonly associated with a pseudo-infarction pattern in the QRS complex, characterized by pathological Q waves or QS complexes in two consecutive leads [22]. This may occur without prior myocardial infarction or echocardiographic akinetic areas and may be observed in up to 70% of CA patients [22,25]. As such, ECG manifestations in patients with CA can vary. AI applications to ECG hold promise for the earlier detection and diagnosis of CA [26].

Early work by Tison et al. [27] utilized a subset of 36,186 ECGs from an academic center database collected over seven years (2010–2017) to employ ML algorithms to estimate various cardiac structure and function parameters, such as left-ventricular mass and left-atrial volume, which are abnormal in CA. Disease detection models were trained to identify four specific cardiac conditions, including CA, based solely on patient ECG profiles. The model achieved an area under the curve (AUC) of 0.86 for predicting the presence of CA [27]. Predictors for CA included features such as the early portion of the QRS complex from lead aVR, and QRS duration. However, this study had two fundamental limitations: the ML of ECG segmentation was optimized only for normal sinus rhythm, potentially restricting its use in patients with arrhythmias, and was from a single medical center limiting generalizability to broader populations.

In a recent multicenter study, Goto et al. [28] developed an AI-ECG model for detecting CA. The study involved 5495 ECGs in the derivation group, 2247 in the validation group, and 3191 in the test group. The ECG model displayed strong predictive accuracy, with AUCs ranging from 0.85 to 0.91 across cohorts [28]. Subtype analysis revealed consistent effectiveness in differentiating ATTR from AL-amyloidosis. Importantly, the model demonstrated the capability to identify amyloidosis even before clinical diagnosis, achieving AUCs of 0.87 to 0.88 at various time intervals preceding diagnosis. The study’s limitations included the possibility of undiagnosed cases of CA in the control group, which may introduce false labels and impact the model’s performance.

Grogan et al. [11] developed an AI-based tool for detecting CA from standard 12-lead ECG. The dataset comprised 2541 patients with AL or ATTR-CA, matched with 2454 controls based on age and sex. Using a subset of 2997 cases and controls, a deep neural network was trained to predict CA presence. The internal validation set (n = 999) and a holdout testing set (n = 999) demonstrated an AUC of 0.91 and a positive predictive value of 0.86 [11]. Notably, the AI model predicted CA over six months before clinical diagnosis in 59% of patients with pre-diagnosis ECG studies. The study also explored single-lead models, with V5 performing best (AUC 0.86, precision 0.78), and a 6-lead (bipolar leads) model achieving an AUC of 0.90 and precision of 0.85 [11]. The study was limited by being conducted at a single center only and having a degree of uncertainty regarding cardiac involvement in some patients.

A follow-up validation study by Harmon et al. observed the postdevelopmental performance of this model to detect CA in the light of multiple potential confounders [29]. Although the AUC of the validation study (0.84) was slightly lower than that of the original study (0.91), it is still considered to have good performance. The AI-ECG tool also maintained acceptable performance across various subgroups, including age, sex, and race/ethnicity (AUC > 0.81), except for the Hispanic population where the performance was lower (AUC 0.66).

Although these studies are promising for the use of AI-driven ECG models, further validation efforts are needed to assess the generalizability of these findings. The clinical role of these AI tools applied to ECG is to flag patients who have a higher probability of having CA and are not diagnostic tools in themselves. Flags of increased risk on ECG for the presence of CA would then serve as an alert to the clinician to consider whether further evaluation would be warranted in the clinical context.

## 3. AI Applications to Echocardiography in Cardiac Amyloidosis

Echocardiography is generally the initial imaging test for investigating CA (Figure 3B–E) [30]. Extensive previous research has demonstrated the diagnostic and prognostic value of two-dimensional (2D) and Doppler echocardiography for the initial identification of CA [31]. Overall, echocardiographic findings of unexplained increased ventricular wall thickness should raise suspicion of CA when in elderly patients or patients with a family or personal history of amyloidosis. Other hallmark changes may include a small left-ventricular (LV) cavity, valve thickening, bi-atrial enlargement in the setting of restrictive physiology, variable presence of a pericardial effusion, and abnormal global longitudinal strain [10,32,33].

A reduced global longitudinal strain (GLS) with relative apical sparing is a specific pattern that is pathognomic for CA and correlates with poor outcomes [18,30,34,35,36,37,38,39,40]. An apical sparing pattern on the strain was found to have a high sensitivity of 93% and specificity of 82% in differentiating CA from other cardiomyopathies with increased wall thickness [41,42]. However, echocardiographic changes may be subtle early in the disease process or may occur with coexisting processes such as hypertension (HTN) or end-stage renal disease (ESRD), and as such are not specific or definitive for CA. Additionally, with regard to strain measurement, the manual contouring of the LV endocardium may result in significant intra- and inter-operator variability that may affect the accuracy of echocardiography in diagnosing CA patients [31,43]. Furthermore, GLS assessment in patients with atrial fibrillation (AF), which is very common among CA patients, is challenging because of the irregular and variable heart rate [44], which affects the timing and duration of the cardiac cycle. As such, research aimed at the use of AI algorithms for the detection of CA using echocardiography may help identify the disease earlier and help reduce observer variability [31,45].

Accurate segmentation of the left ventricle endocardium is essential for assessing myocardial tissue strain and other cardiac function parameters through echocardiography. Zhuang et al. [46] introduced a novel approach that integrates the ’You Only Look Once, Version 3′ (YOLOv3) model, a real-time object detection algorithm, with the Markov Random Field (MRF) model, a probabilistic graphical model for spatial data dependency. This method is designed to segment left-ventricle ultrasound images precisely and in real-time. It has demonstrated superior segmentation accuracy, robustness, and computational speed in constraint and positioning determination compared to previously implemented methods.

In the previously mentioned study by Goto et al., investigators trained and validated an echocardiography video-based model using the apical four-chamber (A4C) view. This model showed high predictive accuracy of CA with an AUC of 0.96 with similar performances in the external validation cohorts [28]. In the subtype analysis, the model showed higher performance on ATTR-CA compared to AL-CA with an AUC of 0.97 vs. 0.95, respectively [28]. Furthermore, the model was able to distinguish CA from other causes of LVH (hypertrophic cardiomyopathy (HCM), HTN, ESRD) with an AUC of 0.96 [28]. Interestingly, a head-to-head comparison between the model and two expert cardiologists attempting to diagnose CA using the same datasets resulted in the outperformance of the model’s AUC over the human readers [28]. However, this superiority must be interpreted in light of a lack of overall clinical picture judgement, as experts had access to only the echocardiography videos without any other clinical information.

In a retrospective study by Chao et al., investigators studied the ability of (ResNet50), a deep learning model based on transthoracic echocardiography, to differentiate CA as the representative disease of restrictive cardiomyopathy (RCM) from constrictive pericarditis (CP) [47]. A total of 381 patients were identified, with a mean age of (68.7 ± 11.4) years, including 197 (51.7%) with CA and 184 (48.3%) with CP. Using only the standard A4C view, ResNet50 provided excellent performance for the facilitated differentiation of CP from CA with an AUC of 0.97 [47]. Moreover, ResNet50 maintained an AUC of 0.84 in an external validation set, showing the potential generalizability of this model [47]. However, from an ML perspective, the overall training sample size was relatively small, as was the external validation cohort. Interpreting the model performance through Gradient-weighted Class Activation Mapping (GradCAM) suggested the model was focusing on the septum and mitral annulus, which are important in the assessment of CA and associated restrictive physiology [47].

In another single-center study by Li et al., investigators retrospectively studied an echocardiography-based automatic DL framework to differentiate increased LV wall thickness etiologies [48]. A total of 586 patients were used for the final analysis (194 HCM, 201 CA, and 191 HTN/others), with a mean age of 55.0 years. Among the individual view-dependent models used in this study, the A4C model had the best performance (AUC: HCM = 0.94, CA = 0.73, and HTN/other = 0.87) with the final fusion model outperforming all the view-dependent models (AUC: HCM = 0.93, CA = 0.90, and HTN/other = 0.92), indicating the potential of this model in the diagnosis and workup of these pathologies that result in increased left-ventricular wall thickness, including CA [48]. As with the previous study, this study is limited by its single-center and retrospective nature and the possibility of referral bias given that patients were identified at a tertiary referral center; these may limit the potential for the generalization of these results.

These studies nevertheless demonstrate the potential for AI algorithms applied to four chamber views from echocardiography to predict the presence of cardiac amyloidosis, and potentially differentiate it from other differential diagnoses associated with increased left-ventricular wall thickening.

## 4. AI Applications to Cardiac Magnetic Resonance in Cardiac Amyloidosis

Cardiovascular magnetic resonance (CMR) provides an assessment of cardiac structure, function, and myocardial tissue characterization, and thus plays a pivotal role in CA diagnosis [49]. The late gadolinium enhancement (LGE) uptake pattern in CA is generally diffuse or patchy throughout the myocardium [47]. Additionally, progressive continuum of LGE identified in CA (none, subendocardial, and transmural) correlate well with the degree of myocardial infiltration and CA burden and associated increased risk of mortality [50]. The presence of LGE in a meta-analysis had reported sensitivity of 85% and specificity of 92% for CA [51]. In addition, quantifying the extracellular volume (ECV) fraction may provide further assessment of the CA burden and offers the potential early detection of CA before LVH development [52]. Although CMR findings of LGE can be supportive of the diagnosis, a normal CMR does not exclude the presence of CA [8]. More importantly, LGE is contraindicated in patients with severely impaired kidney function, which is relatively common in AL-CA patients. Noncontrast T1 and T2 mapping can overcome this limitation. However, their findings only have high diagnostic accuracy (sensitivity: 85%, specificity: 87%) when the pretest probability is high [53,54,55,56,57].

In a retrospective single-center study, Agibetov et al. used CMR data from 502 patients to train CNNs to recognize imaging patterns associated with CA and subsequently test the ability of this model to automatically diagnose it [58]. Out of the three applied DL techniques (from scratch, feature extraction, and fine-tuning), the fine-tuning model consistently had the best diagnostic accuracy with 94% sensitivity, 90% specificity, and an AUC score of 0.96 [58]. However, most of the CA patients had advanced HF, raising a potential limitation of this model in identifying early or preclinical disease.

Another retrospective single-center study by Eckstein et al. investigated the diagnostic performance of CMR imaging in detecting CA using ML algorithms [59]. The study used multi-chamber strain and cardiac function as input parameters for a 41-feature matrix decision tree and supervised ML algorithms. Under supervised conditions, the support vector machine (SVM) algorithm demonstrated competitive diagnostic accuracies of 87.9% (AUC = 0.960) [59]. This suggests that ML of multi-chamber cardiac strain and function could provide innovative insights for non-contrast clinical decision-support systems in the diagnosis of CA. Aside from the study design limitations of an unmatched retrospective cohort, this study approach remains an unsupervised algorithmic model that may not be applicable in other settings.

Progressive sequential causal generative adversarial network (PSCGAN) is a machine learning model in which two neural networks compete with each other by using deep learning methods to become more accurate in their predictions. PSCGAN was implemented by Xu et al. [60] on plain cine MRI images to synthesize LGE-equivalent images with the automatic segmentation of cardiac tissue simultaneously. The model yielded an overall segmentation accuracy of 97.17% and structural similarity index (SSIM) for the synthesized images of 0.78. Since manual segmentation of diagnosis-related tissues is an essential part of the current ischemic heart disease (IHD) workflow in cardiac radiology, PSCGAN may be an efficient tool for IHD and CA diagnosis, precluding the risks of contrast agent toxicity and minimizing interobserver variability.

## 5. AI Applications to Scintigraphy in Cardiac Amyloidosis

Radionuclide scintigraphy plays a pivotal role in CA diagnosis and determining the appropriate next steps for evaluation. It uses different bone tracers including: 99m technetium (Tc)-labeled 3,3-diphosphono-1,2-propanodicarboxylic acid (DPD), 99mTc-labeled pyrophosphate (PYP), and 99mTc-labeled hydroxymethylene diphosphonate (HMDP) [61]. Quantification of radiotracer uptake intensity is essential and is interpreted using semiquantitative visual analysis.

**Figure 4:**
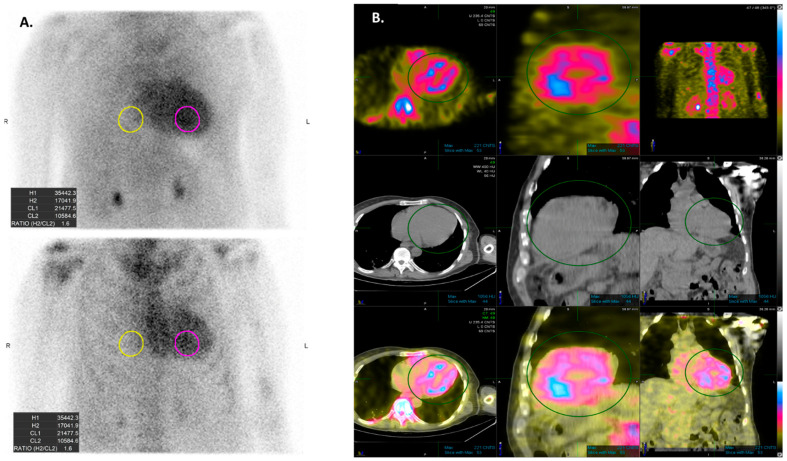
Technetium-labeled cardiac scintigraphy (PYP scan) is commonly used for the diagnosis of cardiac amyloidosis, especially for transthyretin cardiac amyloidosis. (**A**) depicts planar imaging with the use of a semiquantitative evaluation using the heart to contralateral ratio. (**B**) shows SPECT imaging, which allows visualization of the difference in the radiotracer uptake between the left-ventricular blood and the myocardium.

Although a complete evaluation including a clonal analysis of free light chain immunofixation is warranted to differentiate between ATTR-CA and AL-CA, scintigraphy may help differentiate between the subtypes of CA, as the avidity of bone tracers for transthyretin is much higher than that of immunoglobulin light chains (Figure 4) [62]. The explanation for this finding is still under debate, but it may partially be attributed to microcalcifications in cardiac tissue which were found to be more common in ATTR-CA patients [63,64].

The intensity of radiotracer uptake is classified into three grades according to the Perugini staging system (Table 1) [65]. Grade 2 or 3 positive cardiac scintigraphy without any evidence of monoclonal protein has high sensitivity and specificity for ATTR-CA and may establish the diagnosis without histological confirmation [66,67,68]. However, when monoclonal protein is present, the specificity of the test for differentiating amyloidosis subtypes is markedly decreased [69]. Moreover, the specificity of the test is also low in case of Grade < 2 radiotracer uptake, as AL-CA patients may demonstrate low-grade radiotracer uptake [70].

Multiple studies have suggested that embedding AI tools in scintigraphy may increase the accuracy and reliability of radiotracer uptake grading. Furthermore, automated CNN analysis may yield earlier diagnoses of ATTR-CA that could be missed with clinical reading or large-scale screening of scintigraphy images. Halme et al. trained a CNN to automatically detect and classify scintigraphy images [71]. This study compared the performance of two custom-made CNN models to pre-trained CNN state-of-the-art established models implemented in the Keras library, in the detection and classification of cardiac uptake for 1334 patients who underwent HMDP and were visually graded using Perugini grades [71]. The new CNN models had AUC and accuracy of 0.94 and 99%, respectively, for the classification of Perugini grades. Furthermore, the models showed AUC and accuracy of 0.88 and 97%, respectively, in detecting patients with cardiac uptake suggestive of ATTR-CA [71]. The study suggested that implementing AI tools with scintigraphy images may improve the early diagnosis of ATTR-CA.

Delbarre et al. developed a DL model based on a CNN with image-level labels [72]. They studied the performance of this model in two different cohorts: the training dataset and the external validation dataset, which included 3048 and 1633 images, respectively. This model aimed to automatically detect significant cardiac uptake (Perugini grade ≥ 2) on whole-body scintigraphy images [72]. The model achieved a sensitivity of 98.9% and 96.1% for 5-fold cross-validation and external validation, respectively, and a specificity of 99.5% for both 5-fold cross-validation and external validation [72]. Interestingly, the model’s performance was not affected by any confounders that may cause variability in images’ interpretation using conventional grading systems. However, the model was based solely on the Perugini score. Therefore, it cannot differentiate amyloidosis-related and non-amyloidosis-related cardiac uptake, leading to a significant decrease in the positive predictive value. Moreover, the model does not differentiate ATTR-CA from AL-CA, as some AL-CA patients may have grade ≥2 radiotracer uptake. Thus, in real life, the results of this model should be interpreted in the context of the clinical picture [72].

## 6. AI Applications to Pathology in Cardiac Amyloidosis

Clinical and imaging suspicion usually prompt further targeted evaluation for CA. Pathology-related tests, including tissue biopsy (from bone marrow, fat pad, or endomyocardial (Figure 3F)), and serum studies such as monoclonal protein detection (e.g., urine protein immunofixation or serum free light chain ratio analysis) can be used where appropriate for a more definitive diagnosis. Furthermore, laser microdissection with mass spectrometry may provide additional information for subtype analysis differentiating ATTR-CA from AL-CA [73,74]. Significantly, pathology may exclude rare forms of amyloidosis, such as AApoA-1 and AApoA-4 amyloidosis, which is essential for the treatment choice [3,75,76]. However, many of these pathological techniques depend mainly on subjective data interpretation by expert pathologists or may be limited by low diagnostic accuracy (e.g., immunohistochemistry) [77,78]. In addition, tissue acquisition, processing, and interpretation require experience and at unexperienced centers misinterpretation of data can occur. Therefore, utilizing AI models may help decrease observer variability and allow for increased accuracy in pathological evaluation.

Palstrøm et al. [79] applied a Boruta method on a random forest classifier to proteomics data that consisted of 153 laser-dissected amyloid-containing biopsies from a mass spectrometric analysis (75 Congo red positive and 78 Congo red negative). Then, a support vector machine (SVM) learning algorithm was trained to detect specific proteins which were considered “novel amyloid signature” proteins. Examples include clusterin, fibulin-1, vitronectin, C9, and collagen proteins alpha 1–3(VI) chains, in addition to the well-established proteins: apolipoprotein E, apolipoprotein A4, and serum amyloid P [79]. When the trained algorithm was applied to a blinded validation dataset of 103 amyloid-containing biopsies, it performed better in differentiating them from controls, with an accuracy of 1.0. Furthermore, it effectively categorized the patients according to the subtypes in 102 of 103 blinded sets [79].

Though there is a paucity of research in this area, many studies have highlighted the utility of AI on pathological specimens from other non-cardiac tissues (e.g., renal, ligamentum flavum, or corneal tissues). In a study by Kim et al., Raman spectroscopy was used to detect amyloidosis in renal tissue [80]. The authors applied a multivariate analysis approach and an unsupervised ML method to examine the gathered Raman spectroscopic signals. This employed approach successfully detected and classified amyloidosis in renal tissue according to the type and deposition site with accuracy that ranged from 95.6% to 98.4% compared with histopathologic validation [80]. Moreover, the authors stated that this approach could be applied for amyloidosis detection and classification in systemic or hereditary types in other organs of the body [80]. However, the small sample size of this study was a significant limitation. In another recent study, Congo red fluorescence on virtual slides (CRFvs) from 154 renal biopsies was used to build an automated digital pipeline tool to identify the amyloid-containing areas in renal biopsies [81]. Optimum interobserver agreement was observed using this approach (k = 0.90; 95% CI, 0.81–0.98). An improvement in concordance was also revealed when consensus-based CRF vs. evaluation (k = 0.98; 95% CI, 0.93–1) was contrasted with standard Congo red birefringence. Therefore, this study showed good potential for an automated digital pathology pipeline to improve the accuracy of CA diagnosis.

Wang et al. investigated the ability of the ML approach to detect and classify amyloid depositions in histological slides of the ligamentum flavum preceding the progression to systemic amyloidosis [82]. This model was comparable to the gold standard of manual segmentation either in the training (R = 0.98; p = 0.0033) or the application set of histological images (R = 0.94; p = 0.016) [82]. Kessel et al. [83] used corneal tissue sections stained with Congo red, harvested from 42 patients to train and validate a DL algorithm to detect and quantitate the mean amyloid deposits per stromal area. It revealed a sensitivity of 86%, a specificity of 92%, and an F-score of 81 for amyloid recognition [83].

Clinical pathology, including serum studies of CA, is another area of AI application outside of tissue pathology. Utilizing a record of 1075 light chain sequences (428 as from patients with AL amyloidosis, and 647 from healthy subjects), Garofalo et al. [84] presented an ML approach, called LICTOR (λ-LIght-Chain Toxicity predictoR), that can predict the toxicity of monoclonal immunoglobulin light chains in case of systemic light chain amyloidosis. Through analyzing the distribution of somatic mutations in these immunoglobulins, this approach showed a sensitivity of 76%, a specificity of 82%, and an area under the curve of 87% in the prediction of light chains toxicity [84]. Furthermore, LICTOR achieved an accuracy of 83% when applied on an independent group of 12 different sequences of light chains. In a related study by David et al., an ML model using a naïve Bayesian classifier and a weighted decision tree was developed to predict the amyloidogenicity in 143 different immunoglobulin sequences [85]. The naïve Bayesian classifier method revealed prediction accuracies ranging from 60.84% to 81.08%, while the decision trees method resulted in an accuracy of 78% [85]. Previous initial results demonstrate promising strategies for predicting the immunoglobulin sequences’ amyloidogenicity potential. However, future larger scale studies to validate these results are still needed because of the limited sample sizes.

## 7. Discussion and Clinical Implications

Cardiac amyloidosis continues to prove challenging to diagnose, especially early in its clinical course. This may result from several factors such as the considerable phenotypic heterogeneity of the disease and improper patient selection and interpretation of diagnostic tests [86,87,88]. The systemic involvement of comorbidities, particularly in older patients, further complicates reaching an accurate diagnosis [88]. Moreover, issues concerning the expense, accessibility, and invasiveness of specific diagnostic modalities may contribute to the diagnostic challenge. Despite this, it is imperative for physicians to have a high index of suspicion for CA, both for AL-CA, which is considered a hematologic emergency, and ATTR-CA in the era of expanding available therapies. Current diagnostic modalities, including cardiac imaging and pathological testing, have limitations, so the need for a more robust diagnostic process is paramount. The ongoing research and development of AI applications into the diagnostic modalities of CA holds significant potential for the increased accuracy and early detection of CA.

Multiple AI-ECG models accurately detected CA, with AUCs ranging from 0.85 to 0.91 [27,28]. Furthermore, AI-ECG models are able to predict CA over six months before clinical diagnosis [11]. Similarly, AI–echocardiography models are highly accurate, with AUCs ranging from 0.84 to 1.00 [28,47,48]. In one study, an AI–echocardiography model outperformed human experts in differentiating CA from other conditions [28]. AI-CMR models showed promising accuracy in diagnosing CA, reaching AUCs of 0.96 [58,59]. While studies directly comparing AI and human experts in CMR are lacking, the high AUCs achieved using AI may be comparable to skilled radiologists. AI–scintigraphy models also showed promise in aiding the diagnosis of CA, particularly ATTR-CA, with high accuracy (AUCs of 0.88–0.94) and sensitivity (96.1–98.9%) [71,72]. Finally, AI–pathology models have reported diagnostic potential with a model differentiating amyloid-containing biopsies from controls with 100% accuracy, improving inter-observer variability [79]. Moreover, some models could detect pre-symptomatic amyloidosis in ligamentum flavum, while others could predict the toxicity of light chains in AL amyloidosis with reasonable accuracy [82]. In summary, these AI models may change the landscape of CA’s diagnostic evaluation and surveillance, not only by facilitating earlier detection but also by reducing work-up expenses and the need for invasive diagnostic modalities [9,89,90].

While the studies exploring AI applications in various modalities for diagnosing CA showcase promising results, several limitations call for cautious interpretation and further research. A recurring constraint across most studies is the limited sample size, particularly in single-center designs. This raises concerns about the scalability and generalizability of the findings to broader and more diverse populations [91]. One potential approach to this limitation is federated learning, which entails using data from various institutions for AI model training without sharing raw data. This approach has demonstrated significant success in developing AI models for hypertrophic cardiomyopathy detection [91]. Furthermore, many current AI studies are retrospective in nature, hindering the ability to definitively assess the impact of AI impact on early diagnosis and patient outcomes in real-time clinical settings. In addition, some studies solely rely on established scoring systems like Perugini grades, limiting their ability for subtype analysis and potentially impacting positive predictive values. Another limitation is deficient algorithm transparency as many AI models, especially DL algorithms are often considered “black boxes” due to their lack of interpretability. This can be a significant barrier in clinical settings, where understanding the decision-making process of the AI is crucial for trust and adoption by healthcare professionals [92]. The lack of head-to-head comparisons between AI models and standard diagnostic tools across different modalities makes it challenging to establish AI’s superiority in routine clinical practice. Lastly, AI development benefits from larger datasets, but considering the limited amyloid volumes across individual centers, computer modeling may be limited in certain imaging modalities.

## 8. Conclusions

Despite the challenges in diagnosing cardiac amyloidosis (CA), artificial intelligence (AI) is emerging as a game-changer across various diagnostic modalities. AI models applied to ECG, echocardiography, CMR, scintigraphy, and pathology show promise in boosting diagnostic accuracy, enabling earlier detection of CA, and potentially reducing costs. These AI-powered tools have the potential to revolutionize CA diagnosis by facilitating earlier intervention and reducing reliance on expensive or invasive procedures. However, limitations exist, including small study sizes, retrospective data, and a lack of transparency in AI decision-making. Further research is needed to ensure the generalizability of these findings, assess the real-world impact on patient outcomes, and build trust for its regular use among healthcare professionals. Overall, AI presents a powerful new tool in the fight against CA, but further development and validation are crucial before widespread clinical adoption.

## Figures and Tables

**Table 1:** Perugini visual scoring of cardiac uptake.

**Grade 0**	No cardiac uptake.
**Grade 1**	Mild cardiac uptake, less than that in ribs.
**Garde 2**	Moderate cardiac uptake is similar to that in ribs, but uptake in ribs remains clearly visible.
**Garde 3**	Intense cardiac uptake greater than that in ribs with weak or no signal evident in ribs.

## Data Availability

Data are contained within the article.
